# The Adaptive Morphology of *Bacillus subtilis* Biofilms: A Defense Mechanism against Bacterial Starvation

**DOI:** 10.3390/microorganisms8010062

**Published:** 2019-12-30

**Authors:** Sarah Gingichashvili, Danielle Duanis-Assaf, Moshe Shemesh, John D. B. Featherstone, Osnat Feuerstein, Doron Steinberg

**Affiliations:** 1 Biofilm Research Laboratory, Institute of Dental Sciences, Faculty of Dental Medicine, Hebrew University-Hadassah, Jerusalem 9112001, Israel; danielle.assaf@mail.huji.ac.il (D.D.-A.); dorons@ekmd.huji.ac.il (D.S.); 2 Faculty of Dental Medicine, Department of Prosthodontics, Hebrew University-Hadassah, Jerusalem 9112001, Israel; osnat.feuerstein@mail.huji.ac.il; 3 Department of Food Quality and Safety, Institute for Postharvest Technology and Food Sciences, Agricultural Research Organization (ARO), The Volcani Center, Rishon LeZion 7528809, Israel; moshesh@volcani.agri.gov.il; 4 School of Dentistry, University of California San Francisco, San Francisco, CA 94143, USA; John.Featherstone@ucsf.edu

**Keywords:** biofilm, *Bacillus subtilis*, morphology, stress response

## Abstract

Biofilms are commonly defined as accumulations of microbes, embedded in a self-secreted, polysaccharide-rich extra-cellular matrix. This study aimed to characterize specific morphological changes that occur in *Bacillus subtilis* biofilms under nutrient-limiting growth conditions. Under varying levels of nutrient depletion, colony-type biofilms were found to exhibit different rates of spatial expansion and green fluorescent protein production. Specifically, colony-type biofilms grown on media with decreased lysogeny broth content exhibited increased spatial expansion and more stable GFP production over the entire growth period. By modeling the surface morphology of colony-type biofilms using confocal and multiphoton microscopy, we analyzed the appearance of distinctive folds or “wrinkles” that form as a result of lysogeny broth content reduction in the solid agar growth media. When subjected to varying nutritional conditions, the channel-like folds were shown to alter their morphology; growth on nutrient-depleted media was found to trigger the formation of large and straight wrinkles connecting the colony core to its periphery. To test a possible functional role of the formed channels, a fluorescent analogue of glucose was used to demonstrate preferential native uptake of the molecules into the channels’ interiors which supports their possible role in the transport of molecules throughout biofilm structures.

## 1. Introduction

Bacteria, in a natural setting, routinely encounter a wide variety of stress types such as temperature related (heat shock), oxidative, nitrosative, mechanical, and reductive. To offset sub-optimal growth conditions, bacteria develop a number of different adaptation mechanisms, or “stress responses”. These include, but are not limited to, dormant endospore formation, lowered metabolic rate, altered cell morphology [1], changes in gene expression [2], and protein synthesis.

Resilience to stress is also a “hallmark” of biofilms—organized structures of bacterial colonies that adhere to solid or liquid surfaces, encased in a self-secreted, polysaccharide-rich extra-cellular matrix. Bacterial cells within biofilms display enhanced survival capabilities demonstrated by a notably increased resistance to antimicrobial agents [3,4] in single-species and more so in mixed-species biofilms [5].

*Bacillus subtilis (B. subtilis)*, a Gram-positive model bacterium in biofilm research, is able to form morphologically complex and exceptionally robust colony-type biofilm [6]. This bacterium is considered to be largely non-pathogenic, although it has been linked to food spoilage [7,8]. *B. subtilis* commonly resides in association with plant roots in soil [9], as well as within the human oral cavity [10,11] and gastrointestinal tract [12]. Further contributing to its ability to withstand severe environmental conditions is its ability to sporulate, particularly by forming dormant endospores that are embedded within the biofilm. In terms of biofilm forming capacity, *B. subtilis* exhibits a multitude of morphological phenotypes—from thin, flat microstructures to large, spatially heterogenic colony-type biofilms [13]. *B. subtilis* biofilm formation on solid lysogeny broth (LB) medium was shown by Shemesh et al. [14] to occur when the growth medium is supplemented with glycerol and manganese; the biofilm-promoting effect was demonstrated by the appearance of a robust biofilm phenotype alongside increased extracellular matrix production and biofilm-associated sporulation. Vlamakis et al. [15] provided evidence that the structuring of *B. subtilis* biofilms is achieved by heterogeneous differentiation of cells within the colonies into motile, matrix-producing, and sporulating cells. The specific phenotype of a *B. subtilis* colony-type biofilm is determined by multiple environmental factors including growth medium, temperature, presence of cofactors, oxygen availability, etc.

Nutrient deprivation, a common environmental stress encountered by microorganisms across many landscapes, has profound effects on bacteria. Starvation-induced stress has been shown to have an effect on bacterial surface properties such as hydrophobicity [16]. Phaiboun et al. [17] demonstrated the density-dependent and biphasic survival kinetics of *Escherichia coli* when subjecting the bacteria to prolonged periods of starvation. In another study, where *Vibrio cholerae* was exposed to low-nutrient conditions, it underwent significant changes in cell morphology, transforming into coccoid-shaped cells [1]. Across bacterial species, the “stringent response” is the process during which bacteria boost the synthesis of hyperphosphorylated guanosine nucleotides in response to harsh environmental conditions. In the case of *B. subtilis*, this key process aids in the bacterium’s adaptation to fatty acid depletion [18]. Tam et al. [19] demonstrated the specific starvation responses of *B. subtilis* at the gene expression level—specifically, when bacteria were subjected to ammonium and tryptophan starvation. Panihi et al. [20] were able to induce a xylanase-related expression system in *B. subtilis* grown in synthetic medium that was poor in glucose content.

Bacterial cells are particularly vulnerable to nutrient stress when embedded in biofilms due to the limitations of diffusion-based transport of nutrients. Mature *B. subtilis* biofilms can reach hundreds of micrometers in thickness [13] under favorable growth conditions thus severely limiting diffusion-based transport of nutrients to cells in central areas of colony-type biofilms. This is further supported by our recent study [21] reporting a more rapid decrease in green fluorescent protein (GFP) production of cells located in the central regions of the colony-type biofilm over time, compared to those located on the periphery.

Though *B. subtilis* biofilm has been shown to exhibit a wide range of morphologies, little is known about the functional structures of specific colony morphology. One study suggested that a network of channels, formed in *B. subtilis* biofilms, had the potential to facilitate transport of liquid throughout the colony-type biofilms [22]. Another study demonstrated that mineralized areas within *B. subtilis* biofilms act as diffusion barriers, effectively separating between the inner and peripheral regions of the colony-type biofilms [23]. In this study, we used a quantitative approach to analyze the non-uniform structure of *B. subtilis* colony-type biofilm morphology captured by multiphoton and confocal microscopy. We hereby present our findings that further link the changes that occur in the morphology of *B. subtilis* colony-type biofilms to a functional response that promotes bacterial survival under nutrient-limiting conditions.

## 2. Materials and Methods

### 2.1. Strain and Growth Media

Starter cultures of *B. subtilis* YC161 (*P_spank_-gfp*) [24] were grown in lysogeny broth (LB; 10 g of tryptone (Neogen, Lansing, MI, USA), 5 g of yeast extract (Neogen, Lansing, MI, USA) and 5 g of NaCl per liter and incubated at 37 °C at 150 rpm for five h. The biofilm-promoting LB medium was solidified by the addition of 1.5% (*w*/*v*) agar and supplemented with 1% (*v*/*v*) glycerol and 0.1 mM MnSO_4_ [14]. Diluted or nutrient-depleted growth media, for the starvation assays, were prepared by reducing the amount of LB content in the growth medium, e.g., 50% nutrient availability was achieved by using 1 g instead of the standard 2 g LB (100%) per 100 mL of solvent.

### 2.2. Colony-Type Biofilm Formation

For colony-type biofilm formation, starter cultures were prepared as described above. The 2.5 µL of suspension from the starter culture (O.D. 600 nm = 1) was placed onto agar plates of various LB-based media content. The plates were incubated at 30 °C for a period of three days during which the GFP-labelled colony-type biofilms were visualized under a Nikon SMZ25 microscope with an ORCA-R2 camera (Zeiss LSM510 CLS microscope, Carl Zeiss, Oberkochen, Germany). All images were taken using an objective magnification of 1 and an exposure time of 100 ms.

### 2.3. Native Uptake of Nutrients

Uptake of nutrients by colony-type biofilms grown on agar media with limited nutrient availability was simulated by using 2-NBDG (C_12_H_14_N_4_O_8_) (Invitrogen, Eugene, OR, USA), a fluorescent indicator of glucose uptake into living cells. *B. subtilis* colony-type biofilms (BS3610) were cultured for a period of 48 h on agar media with 50% (1 g/100 mL) LB content. Upon reaching morphological maturity, colonies were removed using PBS and placed onto fresh agar media with embedded 2-NBDG. The colonies were detached undisrupted from the growth medium by introducing liquid PBS in between the colony and agar surface. Once detached, a coverslip glass was inserted between the two and lifted with the macrocolony upon it. After a second culture period (48 h), colonies were visualized under a Nikon SMZ25 microscope with an ORCA-R2 camera (Zeiss LSM510 CLS microscope, Carl Zeiss, Oberkochen, Germany).

### 2.4. Multi-Stain Visualization of Colony-Type Biofilms

#### 2.4.1. Confocal Microscopy

Bacterial biofilms were removed gently from agar surfaces to glass cover slips using PBS (PBS tablets, Merck KGaA, Darmstadt, Germany). The colonies were incubated with 700 µL mixed dye of propidium iodide (PI) stain for labelling dead bacteria and concanavalin A (Con A) Alexa Fluor 647 for labelling extracellular polysaccharides (EPS) for 20 min at RT and afterwards washing using PBS. All images were taken using a Zeiss LSM510 CLS microscope (Carl Zeiss, Oberkochen, Germany). Propidium iodide fluorescence was measured using 543 nm excitation and 570 nm emission. Alexa Fluor 647 was measured using a 650 nm excitation wavelength and 668 nm emission. Three-dimensional views of colony-type biofilms were constructed using ZEN software (ZEN 2.3, blue edition, Carl Zeiss Microscopy GmbH, 2011).

#### 2.4.2. Multiphoton Microscopy

Images of mature biofilms on agar media were visualized using a Nikon A1 MP Multi-Photon microscope (Nikon Instruments, Tokyo, Japan). For each examined channel section, a set of images at various depths in the colony-type biofilm was obtained.

### 2.5. Image Processing

#### 2.5.1. Two-Dimensional (2D) Analysis

MATLAB software (R2018a, MathWorks, Inc., Natick, MA, USA) was used for image data analysis. Images of equal size were converted to grayscale by taking the mean value of three color channels in RGB color space. In such grayscale representation, a strong GFP signal is observed as white pixels in the image, whereas areas with non-viable bacteria or areas with no bacterial presence appear as black pixels. Two-dimensional measurements were performed using NIS Elements software (Nikon Instruments, Tokyo, Japan).

#### 2.5.2. Three-Dimensional (3D) Analysis

Multiphoton microscopy was utilized to obtain multiple images at varying depths within the biofilm of small areas where channels were observed. These sets of images were used to render a 3D structure of channels and parabolic approximation was applied to obtain a volumetric analysis of channel sections. Analysis was performed using proprietary code in conjunction with the Volume Viewer applet of MATLAB software.

### 2.6. Statistical Analysis

The data obtained were analyzed statistically using ANOVA following post-hoc *t*-test with Bonferroni correction. All statistical analyses were performed using Microsoft Excel software. Statistically significant values were determined by a *p*-value of less than 0.05.

## 3. Results

### 3.1. Characteristic Features of Bacterial Growth under Nutrient-Limiting Conditions

**Figure 1:**
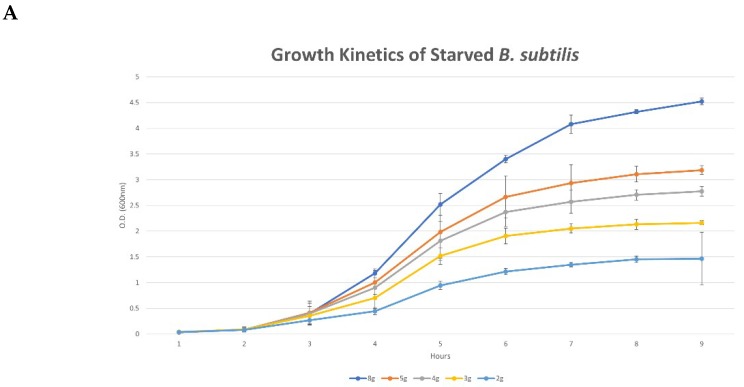

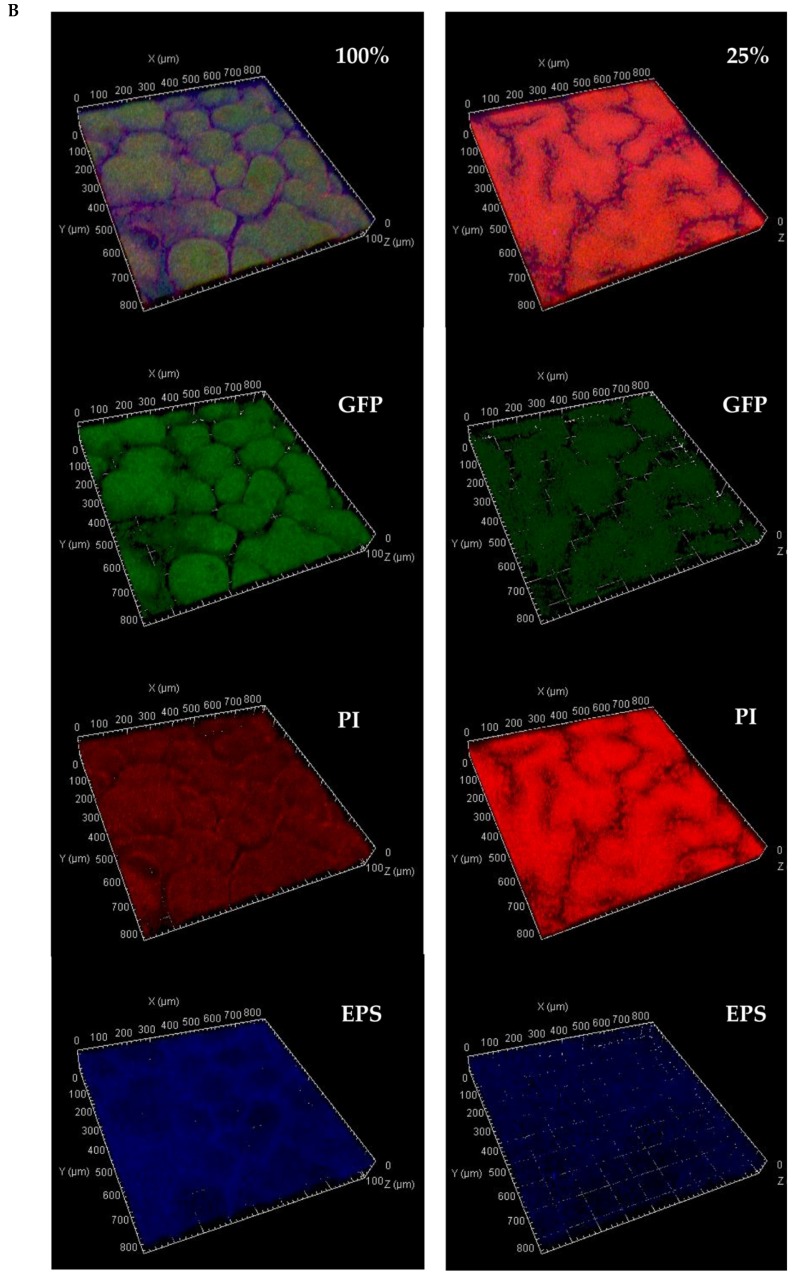

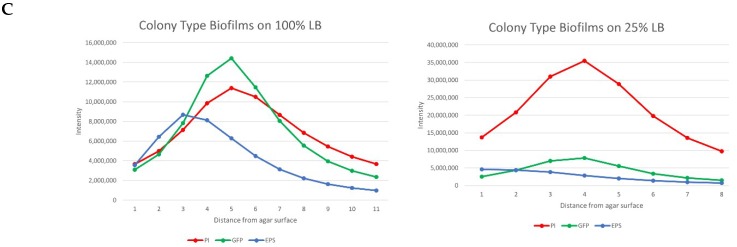
(**A**) OD measurement of *B. subtilis* cells grown under limited nutrient availability. 100% nutrient availability was achieved by medium containing 8 grams of LB per 400 mL of liquid (DDW). The graph curves, top to bottom, represent 8/5/4/3/2 grams of LB per 400 mL DDW, respectively. The results shown are means ± standard deviations (SD) of three independent experiments; (**B**) Confocal imaging of *B. subtilis* biofilm grown either under full (100% (2 g/100 mL), left column) or partial nutrient availability (25% (0.5 g/100 mL), right column). The following stains are shown: green fluorescent protein (GFP, live bacterial cells, second row), propidium iodide (PI, dead bacterial cells, third row), Alexa Fluor 647 (secreted exopolysaccharides, EPS, fourth row). Topmost row shows the combined images (i.e., GFP, PI and EPS); (**C**) Live/dead + EPS depth analysis of confocal images.

Reduction of LB content in liquid growth medium of *B. subtilis* cells results in decreased maximal bacterial concentration reached at the stationary phase of growth (Figure 1A). This decrease was non-linearly correlated to the level of nutrient deprivation—each subsequent dilution of the growth medium by 1 g of LB resulted in an increased effect on maximal optical density (OD) measured. More specifically, when comparing the decreases in maximal OD reached between 5 g to 4 g, 4 g to 3 g, and 3 g to 2 g, we observed a decrease in maximal OD of 41.3%, 61.3%, and 69.3%, respectively.

Confocal microscopy images of mature biofilms (Figure 1B) reveal that peak EPS production was measured at lower layers of the biofilms (i.e., closer to the agar surface) compared to peak GFP and PI signals which were measured closer to the biofilm surface and correlated to live and dead bacterial cells, respectively (Figure 1C). After 48 h, the PI to GFP ratio of starved colony-type biofilms (25% (0.5 g/100 mL) LB content) was on average over four times higher when compared to that of biofilms grown on mediums with 100% (2 g/100 mL) LB content. Extracellular polysaccharides production of starved colonies was on average 54% of the equivalent in non-starved colonies (Figure 1C).

**Figure 2:**
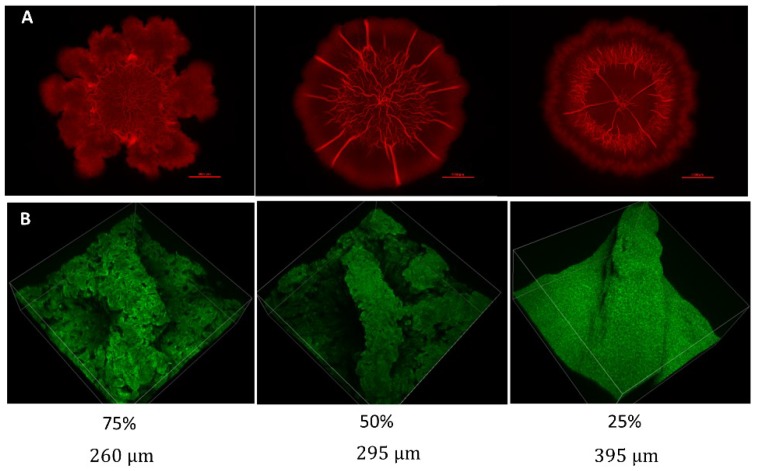
Changes in morphology. (**A**) Effect of LB agar concentration on *B. subtilis* macrocolony morphology. Mature non-nutrient deprived colony (top row, left), colony grown under 50% nutrient availability (top row, middle), 25% nutrient availability (top row, right). Scale bar—5000 μm; (**B**) Effect of progressive LB agar concentration on the structure of a single channel or “wrinkle”. Channel on the surface of a colony grown under 75% nutrient availability (bottom row, right), 50% nutrient availability (bottom row, middle) and 25% nutrient availability (bottom row, right).

**Figure 3:**
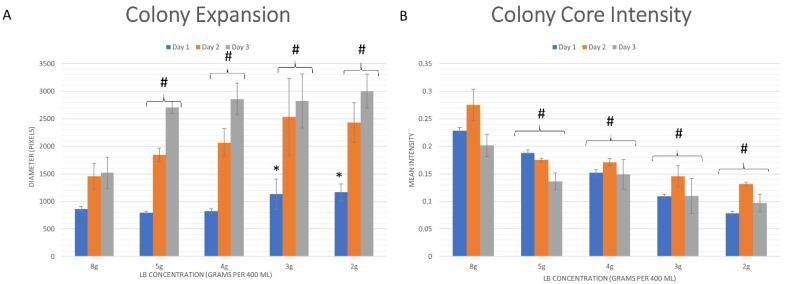
Whole colony expansion-intensity profiles of *B. subtilis* biofilms, grown to maturation over a period of 72 h. (**A**) Colony expansion; (**B**) Colony core intensity. Statistical significance is in relation with the values obtained by colonies grown on non-depleted nutrient medium (8 g LB/400 mL), where * indicates *p* < 0.05, # indicates *p* < 0.01. The results shown are means ± standard deviations (SD) of nine independent experiments.

Colonies grown for a period of three days under some form of nutrient limitation also exhibited several distinct characteristics in their morphology (Figure 2), growth kinetics and long-term GFP production (Figure 3).

Colonies that were subjected to more severe nutrient-limiting conditions exhibited higher expansion rates (i.e., reached further areas of the medium from the point of inoculation) than their non-starved counterparts (Figure 3A). For example, colonies grown on agar with 25% (0.5 g/100 mL) nutrient availability expanded on average 257% (±26%) in diameter compared to 176% (±33%) for colonies grown on agars with 100% (2 g/100 mL) nutrient supply. These differences in expansion rates can be detected as early as 1 day.

The GFP signal intensity produced at the colony core was indicative of the long-term viability (i.e., GFP production) of the cells that comprised that region of the colony-type biofilm (Figure 3B). When grown on biofilm-promoting medium, *B. subtilis* colonies reached a maximal GFP signal at the colony core after approximately 48 h of growth (on day 2); from this time point onwards, GFP production at the core diminished over time. This effect was likewise seen in colonies grown in a nutrient-limited environment, i.e., the GFP signal decreased between day 2 and 3. However, in such colonies, a complementary effect can be observed; when comparing the mean intensity at the core on days 1 and 3, colonies grown on nutrient-limited agar exhibited a net GFP signal increase rather than a decrease seen in their non-starved counterparts. For example, at 100% (2 g/100 mL) nutrient supply, the net difference in GFP signal between day 1 and 3 was a 12% decrease, while the same difference in colonies grown on 25% (0.5 g/100 mL) nutrient agar was an increase of 25%. This effect seems to have been dependent on the level of starvation; the change between a net decrease to a net increase was gradual. At 50% (1 g/100 mL) of nutrient availability, the measured GFP signal was almost equal and differed by a 3% decrease from day 1 to 3.

### 3.2. Quantitative Analysis

#### 3.2.1. Whole Colony Analysis

Figure 2 shows the channels formed on the surfaces of *B. subtilis* colony-type biofilms grown on biofilm-promoting media of varying LB content, assuming different structural and spatial characteristics. Biofilms that were grown on mediums with the standard LB broth content (8 g per 400 mL) developed a complex intercalating network of wrinkles at the colony-type biofilm core with no wrinkles developing on the periphery. In contrast, biofilms grown on media with reduced LB content developed a highly organized network of channels that appeared as direct lines on the surface connecting the colony-type biofilm core to its periphery (Figure 2A). While the number of channels is dependent on the degree of starvation (less channels formed when the LB content was reduced further), their spatial distribution remained such that the angle among neighboring channels was approximately equal.

Adding to the abovementioned differences in the number of channels, multiphoton microscopy reveals an additional distinction in the surface appearance of channels; channels formed by more severely starved colonies appeared to have a smoother, more uniform structure (Figure 2B) than their less starved counterparts.

#### 3.2.2. Volumetric Analysis of Channels

**Figure 4:**
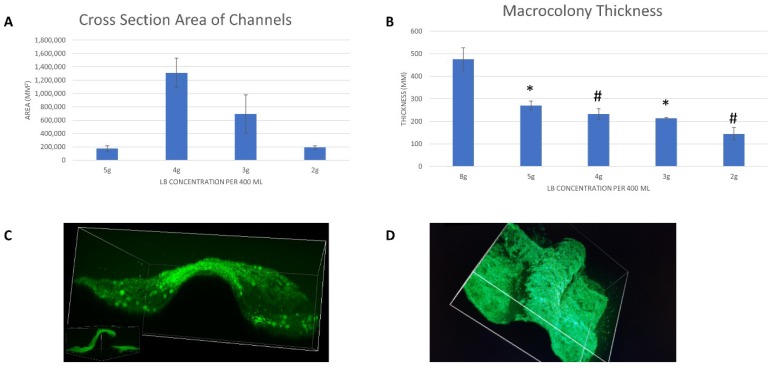
Volumetric analysis of channels. (**A)** Cross-section area of channels, approximated by the integral of a fitted parabola; (**B**) Macrocolony thickness; (**C**) A direct view into a single channel, demonstrating its parabolic structure; (**D**) A top-view of a single channel, at the interface with another channel perpendicular to it. Statistical significance is in relation with the values obtained by colonies grown on non-depleted nutrient medium (8 g LB/400 mL), where * indicates *p* < 0.05, # indicates *p* < 0.01. The results shown are means ± standard deviations (SD) of six independent experiments.

The cross-section of each channel can be approximated from the location of three points that lie in the same plane, perpendicular to the agar surface: the peak of the channel and the two points at which the channel meets the surface of the biofilm that adheres to the agar surface. Those three points allow us to fit the channel curve to a polynomial of a second degree. Figure 4C illustrates the parabolic structure of a typical channel from two different viewpoints, and Figure 4D shows the surface view of the channel, with a visible intersection with another channel, which connects perpendicularly to it. The formulations below use *h* as a measure of individual channel height and *w* for the width of its base.

General formula of a polynomial of a second degree: $f\left(x\right)=ax^{2}+bx+c$.

Coordinates of channel “peak”: $\left(x_{1},f\left(x_{1}\right)\right)=\left(0,h\right)$.

Coordinates of left base point: $\left(x_{2},f\left(x_{2}\right)\right)=\left(-\frac{w}{2},0\right)$.

Coordinates of right base point: $\left(x_{3},f\left(x_{3}\right)\right)=\left(\frac{w}{2},0\right)$.

Polynomial coefficients can then be determined by solving the following system of equations:

(1) $$ax_{1}^{2}+bx_{1}+c=f\left(x_{1}\right)$$

(2) $$ax_{2}^{2}+bx_{2}+c=f\left(x_{2}\right)$$

(3) $$ax_{3}^{2}+bx_{3}+c=f\left(x_{3}\right)$$

Figure 4A illustrates the change in channel cross-section as a function of LB content in the growth medium. The cross-section reached maximum value at 50% (1 g/100 mL) nutrient availability, i.e., 4 g per 400 mL of growth medium. The illustration below demonstrates the mathematical approximation of the cross-section area of an individual channel (shown in gray).

Figure 4B shows the changes that occurred in the colony-type biofilm thickness, disregarding the channels themselves (which rose above the surface thickness). More nutrient availability correlated with an increased measured biofilm thickness, i.e., more biomass.

### 3.3. Co-Localization of 2-NBDG within Channels

**Figure 5:**
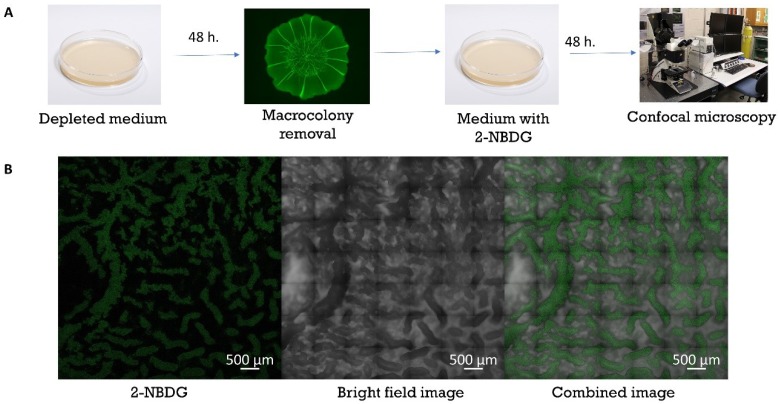
Co-localization of 2-NBDG within channels. (**A**) Experimental design – colony type biofilms, grown to maturation (48 h) on solid mediums with 50% LB content were moved onto growth mediums supplemented with 2-NBDG. Confocal images in (**A**) were taken after an additional period of 48 h; (**B**) Green signal (left) demonstrates the localization of the fluorescent tracer 2-NBDG within the interior of channels (seen as darker areas in the bright field image, right).

Colony-type biofilms, grown to maturation (48 h) on solid media with 50% (1 g/100 mL) LB content, were transferred onto growth media supplemented with 2-NBDG. Confocal images of the colony-type biofilms were taken after an additional period of 48 h. Figure 5B shows the co-localization in the interior of channels (darker areas, right image) with the location of fluorescently tagged molecule (green signal, left image) that was embedded into the solid medium during the later stages of biofilm growth.

## 4. Discussion

This work presents a quantitative analysis of *B. subtilis* colony-type biofilm morphology as it adapted to a reduction in LB content within the growth medium as a model for starvation conditions. We show that reduction of LB content resulted in specific changes in colony-type biofilm morphology, namely, the appearance of channel-like structures that protruded from the center of the colony towards the outer rim. Thus, it is reasonable to assume that these macro-structures essentially formed a physical interface in the form of wrinkles that connected the biofilm’s core to the colony periphery.

### 4.1. Bacterial Kinetics

The morphology of colony-type biofilms has been a significant subject of interest in the field of biofilm research. The morphological diversity of colony-type biofilms formed by *B. subtilis* makes this bacterium a fitting model for research; indeed, several papers have been published revealing formative aspects of biofilm morphology. The formation of wrinkles in *B. subtilis* biofilms was reported to be a consequence of localized cell death [25], mechanical forces [26], and spatial gradients in metabolites [27]. Several beneficial features have been attributed to the wrinkled structure such as structural integrity, elasticity, liquid transport, hydrophobicity, and active protection from other species infiltration [28]. This paper addresses a possible role that the wrinkled architecture plays in *B. subtilis* colony-type biofilms grown in conditions of nutritive stress.

Reduced nutrient availability, modeled by a reduction in carbon sources, has been shown by Zhang et al. [29] to be a positive inducer of EPS production in *B. subtilis* biofilms. This uptick in EPS production was shown by Seminara et al. [30] to drive an increase in bacterial surface motility which they suggest is necessary to overcome the limit of diffusion-based nutrient uptake. This paper supports the findings by directly linking a decrease in the LB content with an increased spatial expansion of *B. subtilis* colony-type biofilms (Figure 3A); under nutrient-limiting growth conditions, *B. subtilis* colony-type biofilms tend to expand spatially without developing a wrinkled structure characteristic to colonies grown on standard biofilm-promoting agar, at their core.

Similar to colonies grown on agar that lacks glycerol and manganese (non-biofilm-promoting, [21]), the nutrient-deprived colonies also demonstrated a more stable bacterial GFP signal production at their core with a smaller characteristic decrease of GFP signal over time (Figure 3B). In both cases, the loss of complex wrinkled formations at the colony core was accompanied by a smaller decrease in the GFP signal produced by the bacterial cells at the colony core, supporting a previous suggestion that there is an inherent “cost” to the formation of the network of wrinkles in the long term [21].

Confocal microscopy images reveal a significantly higher rate of dead bacterial cells at the colony core, accompanied by a considerable reduction in EPS production (Figure 1B). This is consistent with the lack of wrinkled formations at the colony core, as these were replaced by long folds that traversed the colony-type biofilms on the periphery.

*B. subtilis* colony-type biofilms that were grown under nutrient-limiting conditions were shown to have higher spatial expansion rates on solid agar media. While colonies that were grown on standard LB medium (100% or 2 g/100 mL nutrient availability) maintained a stronger GFP signal throughout the growth period, starved colonies were characterized by a reduced but more constant rate of GFP signal production, one that did not suffer from a comparable decrease at the colony core. This might suggest that nutrient-depleted colonies preferentially and actively expand—a proposition that is further supported by the results from the mixed-plate experiments, which showed that the starved colony-type biofilms consistently maintained a significantly higher slope in terms of area occupied by the colony during the growth period.

### 4.2. Biofilm Morphology

This paper demonstrates a formation of channels of a specific morphology and spatial distribution in *B. subtilis* colony-type biofilms grown on solid agar media of depleted LB content. It is important to note that such networks of channels are not limited to colony-type biofilms that are grown under nutrient-limiting conditions. *B. subtilis* biofilms, for example, when grown under biofilm-promoting conditions, develop a complex network of such wrinkles but only at the colony core, not the periphery. Under standard biofilm-promoting conditions, the wrinkled structure is a reflection of the biofilm’s elasticity [21]. Conversely, the channels that develop under nutrient-limiting conditions appear straight and span the entire colony from the center to the very edge of the biofilm. Moreover, the channels appear to be evenly distributed throughout the colony-type biofilm, which suggests that each such channel or wrinkle relates to or “governs” a certain localized area of the colony. This seems to be a particularly conserved feature, while the number of channels on colony-type biofilms that were grown under 50% (1 g/100 mL) nutrient availability (8–10) was on average higher than the equivalent on colony-type biofilms that received 25% (0.5 g/100 mL) nutrients (4–5); in both cases, the channels were equally distributed throughout the colony, i.e., the angle between adjacent channels was significantly larger in the latter.

It has been hypothesized that channels that appear throughout *B. subtilis* colony-type biofilms facilitate liquid transport throughout the biofilm [22]. This work attributes a complementary “suction” force to the channels that was demonstrated by the unaided uptake of fluorescent glucose molecules preferentially from the solid agar medium into the channel interior. Volumetric measurements further appeared to suggest that a decrease in nutrient availability during colony maturation by 50% (1 g/100 mL) triggered a significant increase in the cross-section area and, consequently, the volume of the formed channels; at this LB concentration, the channels appeared most pronounced on the biofilm surface. It is important to note that peripheral channels (not to be confused with the wrinkles that formed at the colony core under 100% (2 g/100 mL) LB content in the biofilm-promoting medium) formed at other LB concentrations as well, but at 50% (1 g/100 mL) LB content, they reached a maximum cross-section area. Combined, these characteristics suggest that there may be a higher rate of fluid exchange taking place in the peripheral area of colony-type biofilms that are subjected to specific nutritionally sub-optimal growth conditions.

The preferred localization of 2-NBDG molecules within the interior of the channels models a “native” uptake of nutrients by the colony from its solid growth medium. It has already been suggested by Wilking et al. [22] that the formed wrinkles facilitate liquid transport throughout the colony-type biofilm—the uptake of molecules from within the medium adds a complementary characteristic by which bacterial cells are able to more efficiently “extract” nutrients from their growth medium by way of altering the colony-type biofilm architecture.

## 5. Conclusions

*B. subtilis’* morphological diversity has been well documented in the literature. The biofilms that are formed by this bacterium are complex, three-dimensional colony-type biofilms with clear and detailed architecture. This paper presents an analysis of the specific morphological changes that occur due to the controlled starvation of *B. subtilis* biofilms. The consistency and nature of these changes suggest that they play a role in the survival of biofilms under nutrient stress. The consistent appearance of the channels as direct lines connecting the core to the outside boundary, combined with the observed increase in their volume as starvation conditions become harsher, hints at their role in aiding the colony-type biofilm survival under a limited supply of nutrients. Thus, a possible link between specific biofilm architecture and functionality can be suggested. In order for such a link to be firmly established, however, we would suggest a comparison of nutritive stress tolerance between wild-type *B. subtilis* and mutant strains, defective in the ability to form channel-like structures, i.e., defective in their biomineralization ability.

## References

[1] Baker R.M., Singleton F.L., Hood M.A. (1983). Effects of nutrient deprivation on Vibrio cholerae. Appl. Environ. Microbiol..

[2] Côté J.P., French S., Gehrke S.S., MacNair C.R., Mangat C.S., Bharat A., Brown E.D. (2016). The Genome-Wide Interaction Network of Nutrient Stress Genes in *Escherichia coli*. mBio.

[3] Girard L.P., Ceri H., Gibb A.P., Olson M., Sepandj F. (2010). MIC Versus MBEC to Determine the Antibiotic Sensitivity of Staphylococcus aureus in Peritoneal Dialysis Peritonitis. Perit. Dial. Int..

[4] Sepandj F., Ceri H., Gibb A., Read R., Olson M. (2004). Minimum inhibitory concentration (MIC) versus minimum biofilm eliminating concentration (MBEC) in evaluation of antibiotic sensitivity of gram-negative bacilli causing peritonitis. Perit. Dial. Int..

[5] Lee K.W.K., Periasamy S., Mukherjee M., Xie C., Kjelleberg S., Rice S.A. (2014). Biofilm development and enhanced stress resistance of a model, mixed-species community biofilm. ISME J..

[6] Branda S.S., Gonzalez-Pastor J.E., Ben-Yehuda S., Losick R., Kolter R. (2001). Fruiting body formation by Bacillus subtilis. Proc. Natl. Acad. Sci. USA.

[7] Thompson J.M., Dodd C.E.R., Waites W.M. (1993). Spoilage of bread by bacillus. Int. Biodeterior. Biodegrad..

[8] Pavić S., Brett M., Petrić I., Laštre D., Smoljanović M., Atkinson M., Kovaćić A., Cetinić E., Ropac D. (2005). An outbreak of food poisoning in a kindergarten caused by milk powder containing toxigenic Bacillus subtilis and Bacillus licheniformis. Archiv Fur Lebensm..

[9] Chen Y., Yan F., Chai Y., Liu H., Kolter R., Losick R., Guo J.H. (2013). Biocontrol of tomato wilt disease by *Bacillus subtilis* isolates from natural environments depends on conserved genes mediating biofilm formation: *Bacillus subtilis* and plant biocontrol. Environ. Microbiol..

[10] Yamane K., Ogawa K., Yoshida M., Hayashi H., Nakamura T., Yamanaka T., Tamaki T., Hojoh H., Leung K.P., Fukushima H. (2009). Identification and Characterization of Clinically Isolated Biofilm-forming Gram-positive Rods from Teeth Associated with Persistent Apical Periodontitis. J. Endod..

[11] Jain K., Parida S., Mangwani N., Dash H.R., Das S. (2013). Isolation and characterization of biofilm-forming bacteria and associated extracellular polymeric substances from oral cavity. Ann. Microbiol..

[12] Hong H.A., Khaneja R., Tam N.M., Cazzato A., Tan S., Urdaci M., Brisson A., Gasbarrini A., Barnes I., Cutting S.M. (2009). Bacillus subtilis isolated from the human gastrointestinal tract. Res. Microbiol..

[13] Bridier A., Le Coq D., Dubois-Brissonnet F., Thomas V., Aymerich S., Briandet R. (2011). The Spatial Architecture of Bacillus subtilis Biofilms Deciphered Using a Surface-Associated Model and In Situ Imaging. Driks A, editor. PLoS ONE.

[14] Shemesh M., Chai Y. (2013). A Combination of Glycerol and Manganese Promotes Biofilm Formation in Bacillus subtilis via Histidine Kinase KinD Signaling. J. Bacteriol..

[15] Vlamakis H., Aguilar C., Losick R., Kolter R. (2008). Control of cell fate by the formation of an architecturally complex bacterial community. Genes Dev..

[16] Kjelleberg S., Hermansson M. (1984). Starvation-induced effects on bacterial surface characteristics. Appl. Environ. Microbiol..

[17] Phaiboun A., Zhang Y., Park B., Kim M. (2015). Survival Kinetics of Starving Bacteria Is Biphasic and Density-Dependent. Weitz JS, editor. PLoS Comput. Biol..

[18] Pulschen A.A., Sastre D.E., Machinandiarena F., Crotta Asis A., Albanesi D., de Mendoza D., Gueiros-Filho F.J. (2017). The stringent response plays a key role in *B acillus subtilis* survival of fatty acid starvation: Stringent response in fatty acid starved *Bacillus subtilis*. Mol. Microbiol..

[19] Tam L.T., Eymann C., Antelmann H., Albrecht D., Hecker M. (2007). Global Gene Expression Profiling of *Bacillus subtilis* in Response to Ammonium and Tryptophan Starvation as Revealed by Transcriptome and Proteome Analysis. J. Mol. Microbiol. Biotechnol..

[20] Panahi R., Vasheghani-Farahani E., Shojaosadati S.A., Bambai B. (2014). Induction of Bacillus subtilis expression system using environmental stresses and glucose starvation. Ann. Microbiol..

[21] Gingichashvili S., Duanis-Assaf D., Shemesh M., Featherstone J.D.B., Feuerstein O., Steinberg D. (2017). Bacillus subtilis Biofilm Development—A Computerized Study of Morphology and Kinetics. Front. Microbiol..

[22] Wilking J.N., Zaburdaev V., De Volder M., Losick R., Brenner M.P., Weitz D.A. (2013). Liquid transport facilitated by channels in Bacillus subtilis biofilms. Proc. Natl. Acad. Sci. USA.

[23] Keren-Paz A., Brumfeld V., Oppenheimer-Shaanan Y., Kolodkin-Gal I. (2018). Micro-CT X-ray imaging exposes structured diffusion barriers within biofilms. NPJ Biofilms Microbiomes.

[24] Chai Y., Norman T., Kolter R., Losick R. (2011). Evidence that metabolism and chromosome copy number control mutually exclusive cell fates in Bacillus subtilis. EMBO J..

[25] Asally M., Kittisopikul M., Rué P., Du Y., Hu Z., Çağatay T., Robinson A.B., Lu H., Garcia-Ojalvo J., Süel G.M. (2012). Localized cell death focuses mechanical forces during 3D patterning in a biofilm. Proc. Natl. Acad. Sci. USA.

[26] Trejo M., Douarche C., Bailleux V., Poulard C., Mariot S., Regeard C., Raspaud E. (2013). Elasticity and wrinkled morphology of Bacillus subtilis pellicles. Proc. Natl. Acad. Sci. USA.

[27] Stewart P.S., Franklin M.J. (2008). Physiological heterogeneity in biofilms. Nat. Rev. Microbiol..

[28] Molina-Santiago C., Pearson J.R., Navarro Y., Berlanga-Clavero M.V., Caraballo-Rodriguez A.M., Petras D., García-Martín M.L., Lamon G., Haberstein B., Cazorla F.M. (2019). The extracellular matrix protects Bacillus subtilis colonies from Pseudomonas invasion and modulates plant co-colonization. Nat. Commun..

[29] Zhang W., Seminara A., Suaris M., Brenner M.P., Weitz D.A., Angelini T.E. (2014). Nutrient depletion in *Bacillus subtilis* biofilms triggers matrix production. New J. Phys..

[30] Seminara A., Angelini T.E., Wilking J.N., Vlamakis H., Ebrahim S., Kolter R., Weitz D.A., Brenner M.P. (2012). Osmotic spreading of Bacillus subtilis biofilms driven by an extracellular matrix. Proc. Natl. Acad. Sci. USA.

